# Seasonal influenza, its complications and related healthcare resource utilization among people 60 years and older: A descriptive retrospective study in Japan

**DOI:** 10.1371/journal.pone.0272795

**Published:** 2022-10-03

**Authors:** Yuriko Hagiwara, Kazumasa Harada, Joshua Nealon, Yasuyuki Okumura, Takeshi Kimura, Sandra S. Chaves

**Affiliations:** 1 Medical Evidence Generation, Medical Affairs, Sanofi, Tokyo, Japan; 2 Department of Health Economics and Outcomes Research, Graduate School of Pharmaceutical Sciences, The University of Tokyo, Tokyo, Japan; 3 Department of Cardiology, Tokyo Metropolitan Geriatric Hospital and Institute of Gerontology, Tokyo, Japan; 4 Medical Evidence Generation, Medical Affairs, Sanofi, Lyon, France; 5 Li Ka Shing Faculty of Medicine, World Health Organization Collaborating Centre for Infectious Disease Epidemiology and Control, School of Public Health, The University of Hong Kong, Hong Kong Special Administrative Region, China; 6 Research and Analytics Department, Real World Data, Co., Ltd., Kyoto, Japan; 7 The Initiative for Clinical Epidemiological Research, Tokyo, Japan; 8 Department of Modelling, Epidemiology and Data Science, Sanofi Pasteur, Lyon, France; 9 Foundation for Influenza Epidemiology, Fondation de France, Paris, France; University of Copenhagen: Kobenhavns Universitet, DENMARK

## Abstract

Evidence suggests that older people aged ≥65 years and those aged 60–64 years with chronic medical conditions are at higher risk of developing severe complications due to influenza virus infection when compared with young, healthy adults. Although seasonal influenza is monitored through a nationwide passive surveillance in Japan, influenza related outcomes and medical resource consumption have not been fully documented. This retrospective database study aimed to describe the epidemiological and clinical characteristics of medically attended influenza cases aged ≥60 years and the associated medical resource consumption in Japan. We used clinically diagnosed influenza (CDI) based on the international classification of disease codes, and laboratory-confirmed influenza (LCI) based on influenza test results, to identify the patient population during a total of nine seasons (2010/2011 to 2018/2019). A total of 372,356 CDI and 31,122 LCI cases were identified from 77 medical institutions. The highest numbers of medically-attended influenza episodes were in patients aged 65–74 years and 75–84 years. On average, across seasons, 5.9% of all-cause hospitalizations were attributable to CDI and 0.4% were LCI. Influenza viruses type A and B co-circulated annually in varying degree of intensity and were associated with similar level of complications, including cardiovascular-related. Oxygen therapy increased with age; by contrast, mechanical ventilation, dialysis, blood transfusion, and intensive care unit admission were higher in the younger groups. In-hospital mortality for inpatients aged ≥ 85 years with CDI and LCI were 18.6% and 15.5%, respectively. Considering the burden associated with medically-attended influenza in this population, influenza prevention, laboratory confirmation and clinical management should be emphasized by general practicians and specialists like cardiologists to protect this aging population.

## Introduction

Every year, seasonal influenza causes 3 to 5 million hospitalizations and 290,000–650,000 respiratory deaths, likely placing huge pressure on healthcare systems, particularly during winter outbreaks in temperate countries [1]. Disease burden is disproportionately high among the elderly, the very young, and people with certain chronic diseases who are often targeted for influenza vaccination [2,3]. The elderly people are particularly vulnerable to influenza virus infection due to declining function of the immune system, or immunosenescence, which results in greater susceptibility to infectious disease and reduction in response to vaccination [4], an effect which may be accentuated by the increased prevalence of underlying conditions in this age group. In Japan, influenza vaccination is recommended for (1) adults ≥ 65 years, (2) those aged 60–64 years with dysfunctional heart, kidney, or respiratory organs which cause severe restrictions in their daily living, and (3) those aged 60–64 years with human immunodeficiency virus infection who are not able to perform usual daily living activities because of immunodeficiency [5].

Severe respiratory complications associated with influenza are well recognized [6] and the Japan Respiratory Society has incorporated treatment and management of patients with influenza into their guidelines for the management of pneumonia in adults [7]. The National Institute of Infectious Disease (NIID, Tokyo, Japan) reports regularly on data from national sentinel surveillance sites, but information on laboratory test used, criteria for testing and general demographics of influenza cases are not robust [8] to help defining influenza disease burden in Japan. In addition, real-world evidence describing laboratory-confirmed influenza is scarce and the surveillance system does not capture information on clinical complications associated with influenza. Here we describe the characteristics of laboratory-confirmed and clinically diagnosed influenza patients aged ≥60 years who presented to outpatient and inpatient care settings, and explore associated complications, clinical outcomes and related health resource utilization during hospital stay. These data could inform public health decision-makers on unmet medical needs related to the treatment and prevention of influenza in Japan.

## Materials and methods

### Data source

The Real World Data (RWD) database is an electronic health database including electronic medical records (EMR), claims, and diagnosis procedure combination (DPC) records. It is a unique database system in Japan because it includes laboratory test results for influenza. The database was launched in July 2015 and is maintained by the Health, Clinic, and Education information Evaluation Institute (HCEI, Kyoto, Japan) with the support of the Real World Data Co., Ltd (Kyoto, Japan). As of 2020, the database included about 20 million inpatients and outpatients from 188 medical institutions in Japan (dating back to 2000).

The DPC payment system is a case-mix patient classification system originally developed in Japan for deciding per diem payments for inpatients in acute care hospitals [9]. When it was launched in 2003, 82 academic hospitals adopted the system, and it has been expanded to 1,757 medical facilities by April 2020 [10]. DPC was developed as a payment system but also contributes as a data source in epidemiological, clinical, and health services research [9].

### Data extraction

For this analysis, we extracted EMR data for outpatients including emergency department visits and inpatients aged ≥60 years with any medical records at facilities which contributed results of influenza diagnostic tests to the RWD from September 6, 2010, through September 1, 2019. The study period was decided to exclude the 2009 influenza pandemic period and the recent pandemic of severe acute respiratory syndrome coronavirus 2 (SARS-CoV-2); and to capture 5 years’ of data prior to RWD’s launch, the minimum period for which medical records must be maintained according to Japanese law.

We defined patients with clinically diagnosed influenza (CDI) based on the International Statistical Classification of Diseases and Related Health Problems 10^th^ revision (ICD-10) administrative codes (ICD-10; J09 to J18 and J22), which are associated with influenza and pneumonia [11]. Any of these codes appearing at any position in the patient’s discharge diagnosis list was captured. In addition, we defined patients with laboratory-confirmed influenza (LCI) based on positive influenza test results (including influenza rapid diagnostic test [IRDTs] and reverse transcription polymerase chain reaction [RT-PCR]). IRDTs are part of routine care to diagnose influenza in Japan [12], and the proportion of cases tested by RT-PCR was unfortunately not available. Moreover, not all patients with influenza virus infection would be tested, and the sensitivity of IRDTs varies, especially among the older population [13–15]. We therefore used two different case definitions (i.e., CDI and LCI) to define a possible range of influenza cases as only relying on laboratory confirmation would likely be an under-ascertainment.

We also extracted data on all-cause hospitalizations (any hospitalization recorded in the EMRs) to understand the contribution of CDI and LCI to the overall number of hospitalizations. The dataset of CDI, LCI, and all-cause hospitalization were independently generated, and the same patient encounter can therefore contribute events to >1 cohort at the same time. Patients also contributed to the same cohort more than once in the study period if multiple diagnoses of influenza (CDI or LCI) were identified with a gap of >30 days.

### Definitions and deduplication

The index date was defined as the date of influenza diagnosis based on ICD-10 code in CDI patients or the date of positive influenza test results in LCI patients. A case with two or more diagnoses or positive test results within 30 days from the index date was considered as a single case, type of virus identified from the first test adopted. The index date of all-cause hospitalization was defined as the date of admission.

CDI, LCI, and all-cause hospitalization events were divided based on the index date into a total of 9 seasons (2010/2011 to 2018/2019). In accordance with the definitions used by the NIID [16], the one year between starting in week 36 until week 35 of the following year were defined as the influenza season.

CDI and LCI in each season were further classified into outpatient and inpatient episodes. Patients whose index date occurred during hospitalization or who have an admission record within 14 days after the index date was defined as inpatients; others were considered outpatients.

The presence of comorbidity was defined based on the ICD-10 codes recorded on or any time before index date (S1 Table). The following diseases were evaluated as comorbidities: chronic lung disease, asthma, diabetes, neurological disease, hypertensive disease, chronic heart failure, chronic heart disease, chronic liver disease, chronic kidney disease, end stage renal disease, cancer, and anemia.

Complications that occurred during hospitalization for patients with CDI or LCI were defined as being diagnosed between the day following the index date and the day of discharge. Specifically, diseases listed as complications were: respiratory failure, exacerbations of chronic obstructive pulmonary disease, asthma exacerbation, exacerbations of diabetes, encephalitis, myopathy, myositis, hypertensive disease, acute myocardial infarction, acute heart failure, hypertensive heart failure, myocarditis and pericarditis, hypertensive heart disease, atrial fibrillation or flutter, stroke, and kidney failure (S2 Table).

Use of medical resources such as intensive care unit (ICU), mechanical ventilation, oxygen therapy, dialysis, tube feeding, and blood transfusion were evaluated from the day after the index date (to distinguish between existing comorbidities/treatments and complications arising from influenza) to the date of discharge, using procedure codes in the DPC system (S3 Table).

Influenza was divided into type A, type B, and unknown, according to the influenza test results at the index date. Patients who were infected with both types A and B in the same index date (co-detection) were included in type AB group.

### Analyses

Age was calculated by subtracting the birth year from the year of the index date because only birth years were retained after the data anonymization process. Patients were classified into four age groups (60–64, 65–74, 75–85, and ≥85 years). The standardized measure of weekly CDI and LCI outbreak trends were calculated as the number of CDI and LCI cases per epidemic week divided by the number of all-cause hospitalizations per epidemic week, stratified by season, to adjust for seasonality and changing number of enrolled medical institutions. Trends by age group were compared by Cochran-Armitage test and Spearman’s rank-sum test. The difference in proportion between groups was evaluated by Fisher’s exact test.

As a post hoc analysis, the trend of the seasonal epidemic of CDI and LCI was compared with the data from Hospitalized Influenza Surveillance (HIS) conducted by NIID. HIS annually publishes the number of hospitalizations caused by influenza in patients aged ≥60 years from 500 designated sentinel hospitals (HIS60) since the 2011/2012 season [17–20]. The rate ratio of HIS60 was calculated as the number of HIS60 per season divided by the number of HIS60 in 2014/2015 (the study mid-point reference season, rate ratio = 1.0). The standardized measure of CDI/LCI activity which was calculated by dividing the number of events by the number of all-cause hospitalization per season was also expressed relative to the 2014/2015 season (the reference season).

Two-sided p-values of < 0.05 were considered statistically significant in chi-squared tests for trend. All analyses were performed using R 4.0.2 (R Foundation for Statistical Computing. Vienna, Austria).

### Compliance with ethics guideline

This study was approved by the ethics committee of Tokyo Metropolitan Geriatric Hospital and Institute of Gerontology (Tokyo, Japan. No. R20-26). This article is based on an existing anonymized database and does not contain any studies with human participants or animals.

## Results

### Enrolled medical institutions

From September 6, 2010 through September 1, 2019, among the 188 medical institutions that contributed data to the RWD database, 77 contributed to this analysis through provision of influenza test result and associated clinical data in ≥1 season (Table 1). The characteristics of included health facilities differed from national statistics: for example, medical institutions with 0–19 beds such as clinics are the most common type of institution in Japan (92.5%) but comprised only 11.7% of the institutions in the current study. Larger facilities were therefore over-represented.

**Table 1:** Characteristics of medical institutions included in the study compared to those at the national level in Japan.

		Number of medical institutions			
		Enrolled hospital		In Japan*	
		(n = 77)	(%)	(n = 110,916)	(%)
Number of beds					
	0–19	9	(11.7)	102,616	(92.5)
	20–99	8	(10.4)	2,945	(2.7)
	100–299	23	(29.9)	3,892	(3.5)
	300–499	24	(31.2)	1,062	(1.0)
	500 -	13	(16.9)	401	(0.4)
Regional distribution					
	Hokkaido	4	(5.2)	3,949	(3.6)
	Tohoku	5	(6.5)	7,079	(6.4)
	Kanto	12	(15.6)	35,497	(32.0)
	Chubu	11	(14.3)	17,102	(15.4)
	Kinki	30	(39.0)	22,297	(20.1)
	Chugoku	4	(5.2)	7,300	(6.6)
	Shikoku	2	(2.6)	3,781	(3.4)
	Kyusyu-Okinawa	9	(11.7)	13,911	(12.5)

* Data from a national survey of medical institutions in Japan. [21].

### Study population

The number of medical institutions collaborating data to this analysis fluctuated over the study period from the lowest of 37 facilities in 2010/2011 season to the highest number (69 facilities) in 2017/2018 (Table 2). There was an annual average of 41,373 (SD 17,282) CDI cases, 12-fold higher than the annual average of LCI (3,458). These numbers varied substantially by year and the standardized measure of medically-attended CDI and LCI followed a significant upward trend (p < 0.001, both). The highest numbers of medically-attended influenza episodes were in patients aged 65–74 years and 75–84 years. There was a decreasing proportion of CDI and LCI cases in patients aged 60–64 years, representing 14.7% and 28.7% of events in 2010/2011, declining to 9.0% and 15.4% in 2018/2019 season. On average, across seasons, 5.9% (103,716/1,763,247) of all-cause hospitalizations were attributable to CDI and 0.4% (7,654/1,763,247) were LCI.

**Table 2:** Summary of medical institutions, all-cause hospitalizations, and the frequency of medically-attended patients with clinically diagnosed and laboratory-confirmed influenza by season and age group, from 2010/2011 to 2018/2019.

		2010/2011		2011/2012		2012/2013		2013/2014		2014/2015		2015/2016		2016/2017		2017/2018		2018/2019		Annual average (SD)	
Number of medical institutions [number of DPC hospitals]																					
		37 [35]		40 [37]		44 [41]		51 [46]		58 [51]		65 [56]		68 [57]		69 [56]		67 [55]			
Number of all-cause hospitalization																					
		137,854		148,732		164,522		181,727		203,051		231,148		239,229		234,202		222,782		195,916	(38,956)
Number of clinically diagnosed influenza cases (a total of 372,356)																					
		19,218		21,989		26,141		31,570		41,528		49,730		60,790		61,422		59,968		41,373	(17,282)
		n	(%)	n	(%)	N	(%)	n	(%)	n	(%)	n	(%)	n	(%)	n	(%)	n	(%)		
	Age group (years old)																				
	60–64	2,827	(14.7)	2,927	(13.3)	3,286	(12.6)	3,771	(11.9)	4,558	(11.0)	5,296	(10.6)	5,630	(9.3)	5,671	(9.2)	5,371	(9.0)	4,371	(1,182)
	65–74	5,437	(28.3)	6,273	(28.5)	7,666	(29.3)	9,680	(30.7)	12,447	(30.0)	15,104	(30.4)	17,412	(28.6)	16,926	(27.6)	16,297	(27.2)	11,916	(4,767)
	75–84	6,588	(34.3)	7,718	(35.1)	8,873	(33.9)	10,682	(33.8)	13,840	(33.3)	16,729	(33.6)	20,967	(34.5)	21,167	(34.5)	20,804	(34.7)	14,152	(5,970)
	85-	4,366	(22.7)	5,071	(23.1)	6,316	(24.2)	7,437	(23.6)	10,683	(25.7)	12,601	(25.3)	16,781	(27.6)	17,658	(28.7)	17,496	(29.2)	10,934	(5,436)
Standardized measure of clinically diagnosed influenza cases (%)																					
		13.9		14.8		15.9		17.4		20.5		21.5		25.4		26.2		26.9		20.3	(5.1)
Number of laboratory-confirmed influenza cases (a total of 31,122)																					
		819		1,738		2,548		1,892		3,990		3,368		5,369		6,190		5,208		3,458	(1,860)
		n	(%)	n	(%)	N	(%)	n	(%)	n	(%)	n	(%)	n	(%)	n	(%)	n	(%)		
	Age group (years old)																				
	60–64	235	(28.7)	314	(18.1)	465	(18.2)	439	(23.2)	645	(16.2)	737	(21.9)	768	(14.3)	1,001	(16.2)	803	(15.4)	601	(253)
	65–74	240	(29.3)	510	(29.3)	721	(28.3)	679	(35.9)	1174	(29.4)	1146	(34.0)	1529	(28.5)	2000	(32.3)	1595	(30.6)	1,066	(575)
	75–84	203	(24.8)	564	(32.5)	817	(32.1)	527	(27.9)	1214	(30.4)	949	(28.2)	1604	(29.9)	1788	(28.9)	1598	(30.7)	1,029	(555)
	85-	141	(17.2)	350	(20.1)	545	(21.4)	247	(13.1)	957	(24.0)	536	(15.9)	1,468	(27.3)	1,401	(22.6)	1,212	(23.3)	762	(508)
Standardized measure of laboratory-confirmed influenza cases (%)																					
		0.6		1.2		1.5		1.0		2.0		1.5		2.2		2.6		2.3		1.7	(0.7)

DPC, diagnosis procedure combination.

The total number of CDI that occurred in outpatient settings during the study period was 268,640, more than 2-fold higher than inpatient CDI episodes (Table 3). There were 23,468 outpatient LCI cases and 7,654 inpatients. The number of inpatients with CDI and LCI increased with age. For outpatients, influenza episodes peaked at a younger age (65–74 or 75–84) before declining. The prevalence of comorbidities was similar across groups, but slightly elevated in inpatients, with diabetes and cardiovascular diseases such as hypertensive disease and chronic heart disease being the most common comorbidities. The proportion of participants without comorbidities was higher (more than 2-fold) in outpatients compared with inpatients in either CDI or LCI.(Table 3).

**Table 3:** Characteristics of medically-attended patients with clinically diagnosed and laboratory-confirmed influenza by care setting, from 2010/2011 to 2018/2019.

		Clinically diagnosed influenza				Laboratory-confirmed influenza			
Characteristics		Outpatient		Inpatient		Outpatient		Inpatient	
		(n = 268,640)		(n = 103,716)		(n = 23,468)		(n = 7,654)	
Age group (years)		n	(%)	n	(%)	n	(%)	n	(%)
	60–64	32,897	(12.2)	6,440	(6.2)	4,982	(21.2)	425	(5.6)
	65–74	84,224	(31.4)	23,018	(22.2)	8,043	(34.3)	1,551	(20.3)
	75–84	90,595	(33.7)	36,773	(35.5)	6,487	(27.6)	2,777	(36.3)
	≥85	60,924	(22.7)	37,485	(36.1)	3,956	(16.9)	2,901	(37.9)
Comorbidities									
	Chronic lung disease	99,921	(37.2)	35,697	(34.4)	6,374	(27.2)	2,448	(32.0)
	Asthma	44,219	(16.5)	13,724	(13.2)	2,876	(12.3)	1,045	(13.7)
	Diabetes	89,442	(33.3)	40,734	(39.3)	6,135	(26.1)	2,716	(35.5)
	Neurological	19,985	(7.4)	14,245	(13.7)	1,368	(5.8)	986	(12.9)
	Hypertensive disease	121,327	(45.2)	57,905	(55.8)	9,362	(39.9)	4,267	(55.7)
	Chronic heart failure	53,058	(19.8)	30,038	(29.0)	2,995	(12.8)	1,914	(25.0)
	Chronic heart disease	127,000	(47.3)	61,444	(59.2)	7,958	(33.9)	4,044	(52.8)
	Chronic liver disease	45,511	(16.9)	19,265	(18.6)	3,324	(14.2)	1,160	(15.2)
	Chronic kidney disease	22,673	(8.4)	13,602	(13.1)	1,464	(6.2)	970	(12.7)
	End stage renal disease	3,416	(1.3)	1,745	(1.7)	249	(1.1)	134	(1.8)
	Cancer	65,226	(24.3)	32,945	(31.8)	3,694	(15.7)	1,925	(25.2)
	Anemias	35,572	(13.2)	20,082	(19.4)	2,236	(9.5)	1,258	(16.4)
	Without comorbidities	45,044	(16.8)	7,428	(7.2)	7,912	(33.7)	875	(11.4)

### Seasonal trends of inpatients with CDI and LCI among all-cause hospitalizations

**Fig 1:**
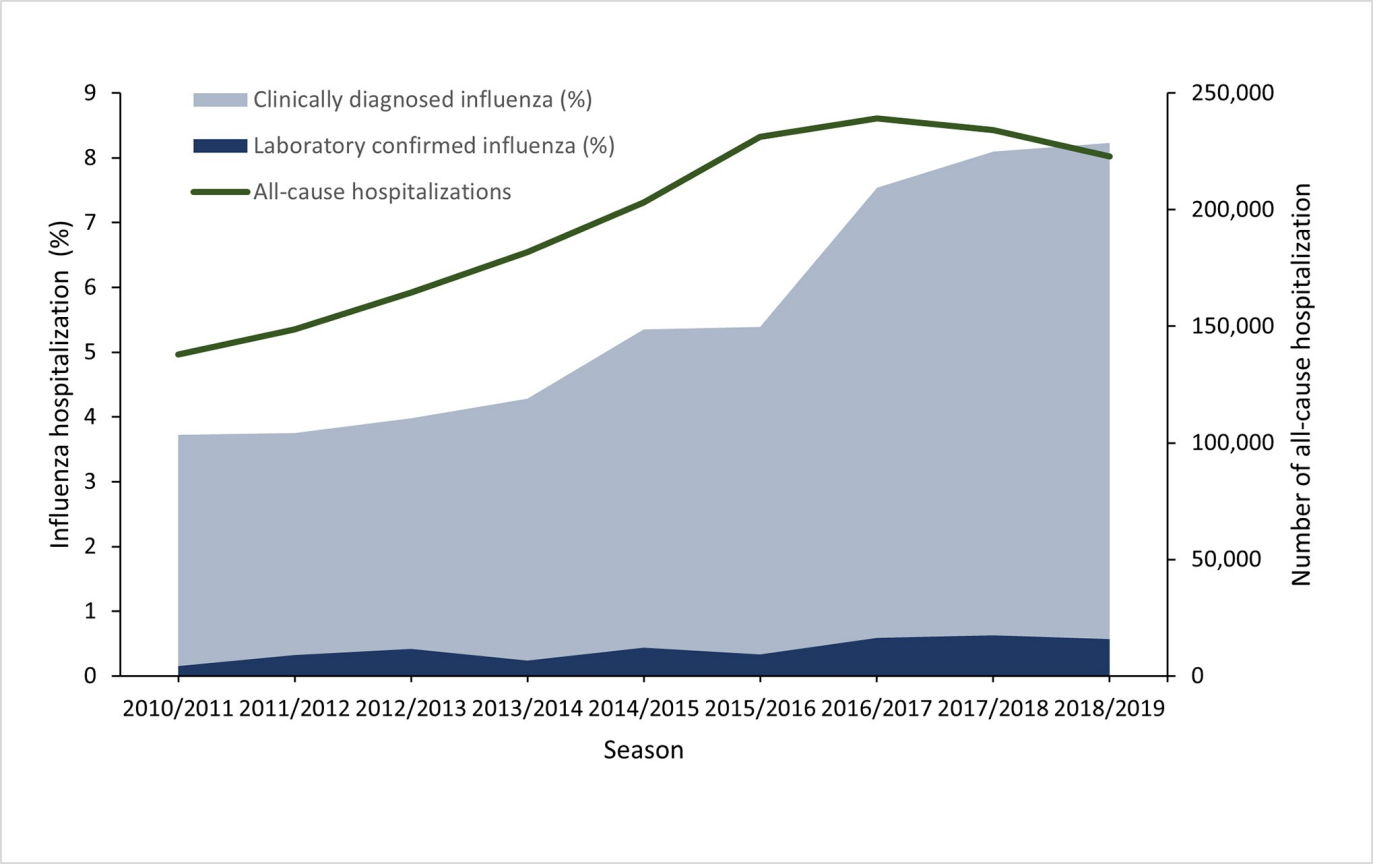
The number of all-cause hospitalizations and proportion of hospitalizations for clinicaly diagnosed influenza and laboratory-confirmed influenza. The percentage refers to the number of clinically diagnosed influenza and laboratory-confirmed influenza cases divided by the total number of all-cause hospitalizations recorded for that season.

**Fig 2:**
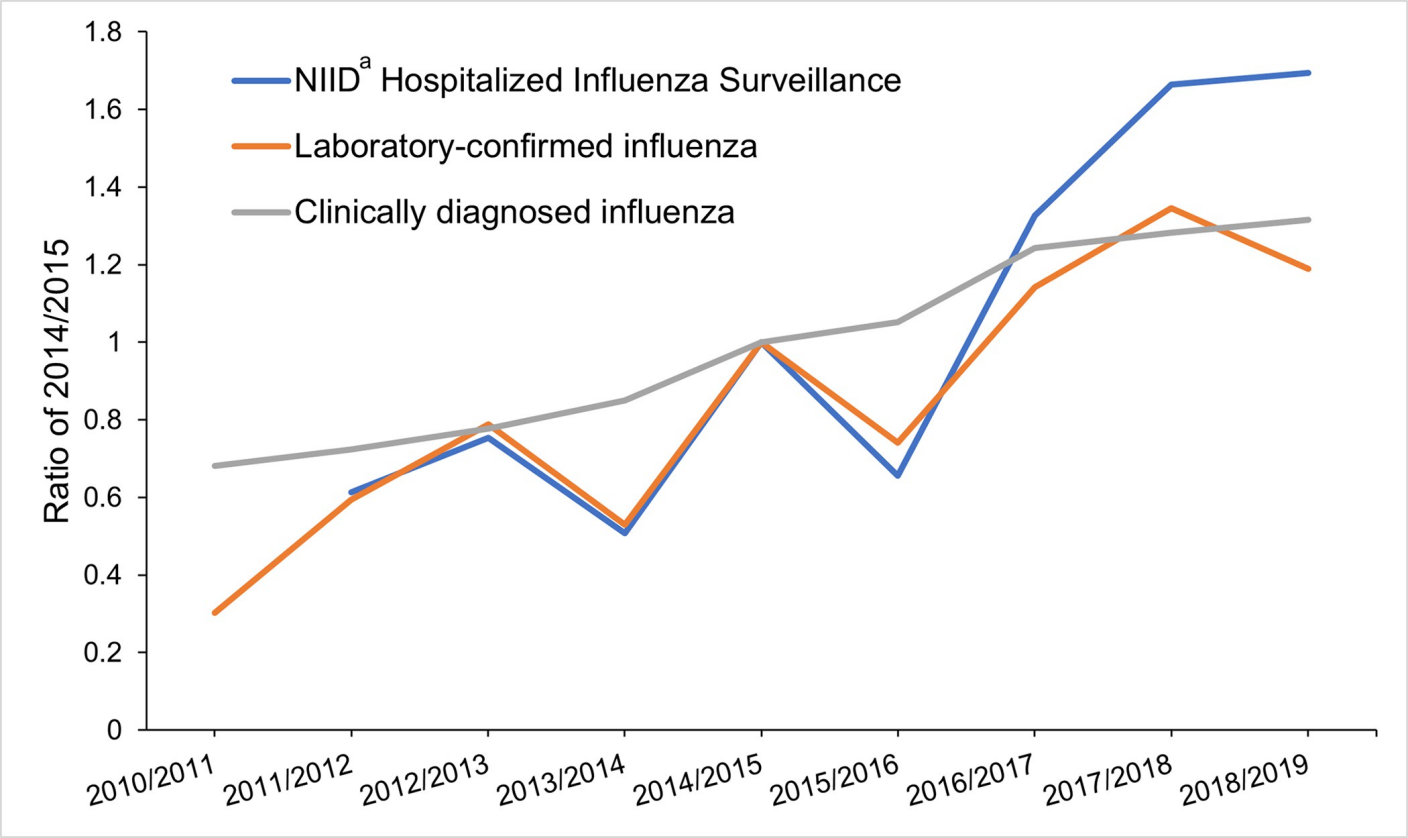
Trends of clinically diagnosed influenza and laboratory-confirmed influenza from our study compared with national data on influenza hospitalizations among ≥60 years reported from the National Institute of Infectious Disease. The ratio of the standardized measure of clinically diagnosed influenza (CDI) and laboratory-confirmed influenza (LCI), and the rate of influenza-hospitalizations among aged ≥60 years hospitalizations in reported by the NIID^a^ Hospitalized Influenza Surveillance (HIS) divided by value 2014/2015 (the reference, 1.0). CDI and LCI are derived from our dataset; HIS represent data from the National Institute of Infectious Disease in Japan. Hospitalizations estimated those ≥60 years in both numerators and denominators. ^a^National Institute of Infection Disease.

The number of all-cause hospitalizations increased from 137,854 in 2010/2011 to 222,782 in 2018/2019. The percentage of inpatients with CDI and LCI among all-cause hospitalizations increased annually from 3.7% and 0.2% in 2010/2011 to 8.2% and 0.6% in 2018/2019, respectively (Fig 1). The rate of standardized measure of CDI and LCI cases in the current study were consistent with estimates from the HIS data except for the three seasons (2010/2011, 2017/2018, and 2018/2019) (Fig 2).

### Type of influenza infections among medically-attended patients with LCI

**Fig 3:**
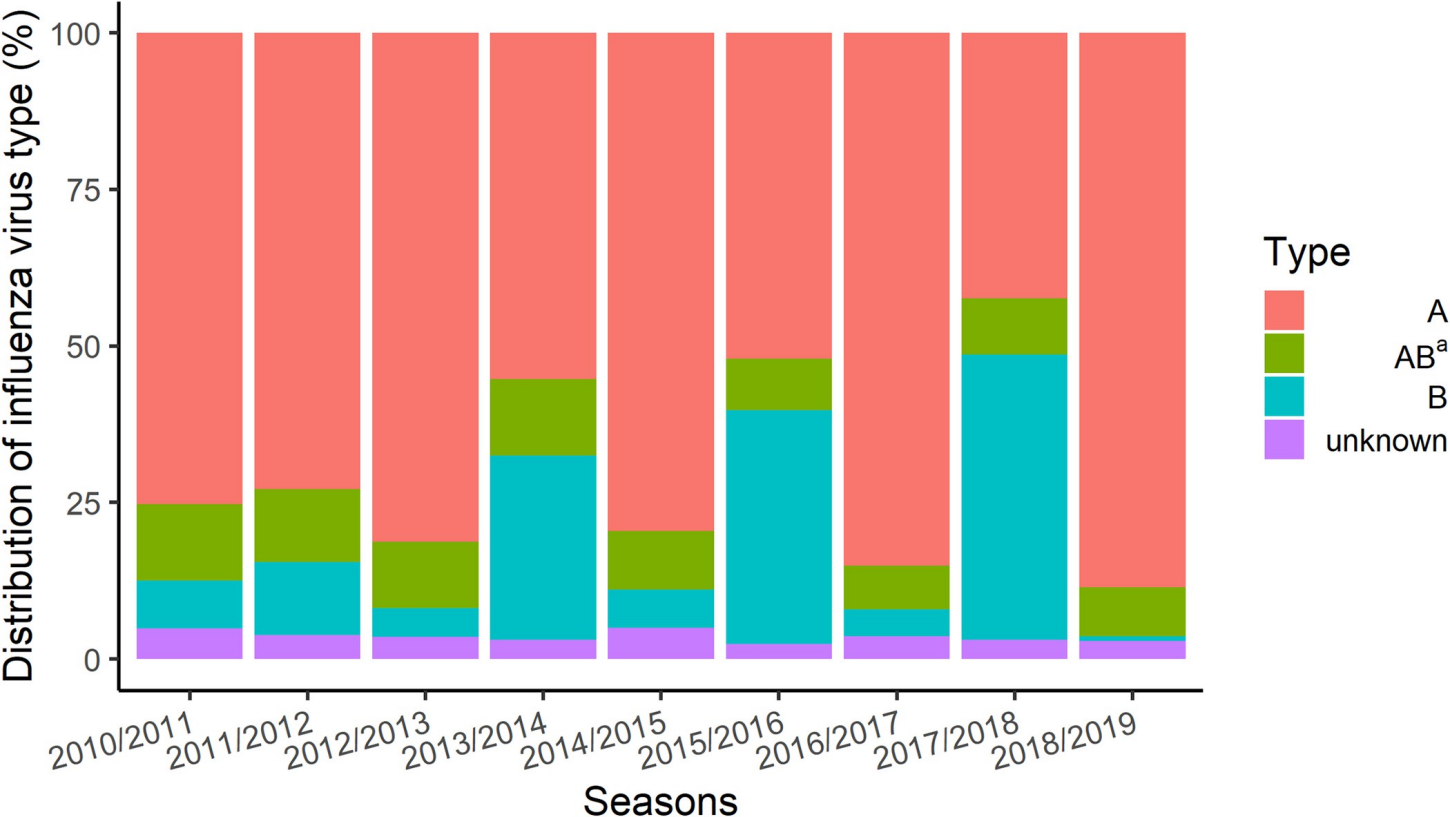
Distribution of influenza virus type among medically-attended patients with laboratory-confirmed influenza, by influenza season (2010/2011–2018/2019). ^a^AB; Patients who recorded with both types A and B positive influenza test result on the same inedex date (co-detection).

Of 31,122 outpatients and inpatients with LCI, 24,515 (78.8%); 8,321 (26.7%); and 1,075 (3.5%) were type A, type B, and unknown, respectively. The percentage of type B cases was highest during 2013/2014 (41.6%), 2015/2016 (45.5%), and 2017/2018 (54.5%) seasons. Co-detection of type A and B occurred on average among 9.8% of the cases, which range from 7.0% to 12.3%. (Fig 3, S4 Table).

### Trend of influenza by season

**Fig 4:**
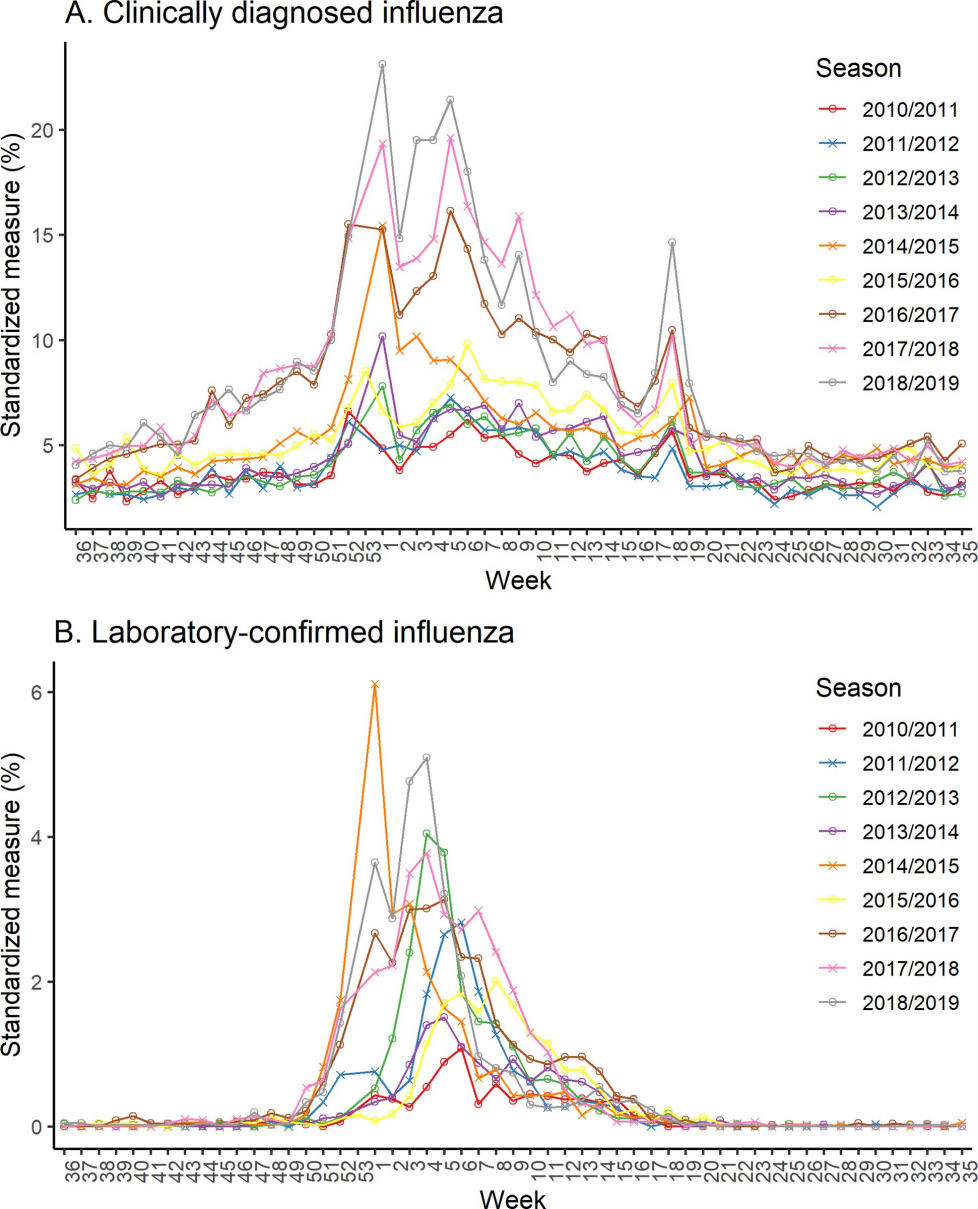
Time course of influenza epidemic in clinically diagnosed and laboratory-confirmed influenza among all-cause hospitalizations by season from the 2010/2011–2018/2019. **(**A) clinically diagnosed influenza (CDI) (B) laboratory-confirmed influenza (LCI). The standardized measure was calculated by dividing the number of inpatient and outpatient with CDI or LCI per week by the number of all-cause hospitalizations per week. Week one started on January 1.

The trend of the weekly CDI cases standardized by all-cause hospitalizations showed a clear seasonal epidemic curve in all observed seasons (Fig 4A). Seasonal epidemics of LCI cases were more noticeable than CDI, normally peaking between weeks 53 and week 8 each season (Fig 4B).

### In-hospital complications and mortality

In CDI and LCI inpatients, the most common complication was myopathy, followed by acute heart failure, hypertensive disease, respiratory failure, stroke, and hypertensive heart failure. The proportion of each of these complications increased with age (test for trend: p <0.001 in CDI and p <0.05 in LCI, Table 4) and almost doubled in the oldest age group compared to those aged 60–64 years.

**Table 4:** Complications and deaths among patients hospitalized with clinically diagnosed and laboratory-confirmed influenza by age group and influenza type, from 2010/2011–2018/2019 seasons.

		Clinically diagnosed influenza								Laboratory-confirmed influenza											
		Age group								Age group								Influenza type^a^			
Patient outcomes		60–64		65–74		75–84		85-		60–64		65–74		75–84		85-		Type A		Type B	
		(n = 6,440)		(n = 23,018)		(n = 36,773)		(n = 37,485)		(n = 425)		(n = 1,551)		(n = 2,777)		(n = 2,901)		(n = 6,062)		(n = 1,760)	
Complications		n	(%)	n	(%)	n	(%)	n	(%)	n	(%)	n	(%)	N	(%)	n	(%)	n	(%)	n	(%)
	Respiratory failure*	439	(6.8)	1,820	(7.9)	3,021	(8.2)	3,480	(9.3)	21	(4.9)	83	(5.4)	159	(5.7)	198	(6.8)	353	(5.8)	111	(6.3)
	Exacerbations of COPD	9	(0.1)	76	(0.3)	95	(0.3)	61	(0.2)	0	(0.0)	6	(0.4)	5	(0.2)	3	(0.1)	10	(0.2)	5	(0.3)
	Asthma exacerbation	24	(0.4)	110	(0.5)	160	(0.4)	125	(0.3)	4	(0.9)	11	(0.7)	12	(0.4)	11	(0.4)	29	(0.5)	8	(0.5)
	Exacerbations of diabetes	9	(0.1)	18	(0.1)	38	(0.1)	23	(0.1)	0	(0.0)	0	(0.0)	1	(0.0)	1	(0.0)	1	(0.0)	1	(0.1)
	Encephalitis	33	(0.5)	101	(0.4)	108	(0.3)	47	(0.1)	1	(0.2)	5	(0.3)	3	(0.1)	1	(0.0)	7	(0.1)	3	(0.2)
	Myopathy*	411	(6.4)	2,004	(8.7)	4,869	(13.2)	6,614	(17.6)	31	(7.3)	112	(7.2)	316	(11.4)	410	(14.1)	690	(11.4)	199	(11.3)
	Myositis	4	(0.1)	12	(0.1)	18	(0.0)	11	(0.0)	0	(0.0)	1	(0.1)	3	(0.1)	0	(0.0)	3	(0.0)	0	(0.0)
	Hypertensive disease*	448	(7.0)	1,818	(7.9)	3,350	(9.1)	3,612	(9.6)	23	(5.4)	82	(5.3)	157	(5.7)	202	(7.0)	371	(6.1)	95	(5.4)
	Acute Myocardial Infarction	54	(0.8)	246	(1.1)	482	(1.3)	450	(1.2)	4	(0.9)	13	(0.8)	17	(0.6)	28	(1.0)	58	(1.0)	7	(0.4)
	Acute Heart Failure*	432	(6.7)	1,579	(6.9)	3,207	(8.7)	4,387	(11.7)	21	(4.9)	76	(4.9)	147	(5.3)	233	(8.0)	377	(6.2)	110	(6.3)
	Hypertensive Heart Failure*	263	(4.1)	1,034	(4.5)	2,221	(6.0)	3,008	(8.0)	14	(3.3)	46	(3.0)	93	(3.3)	151	(5.2)	237	(3.9)	68	(3.9)
	Myocarditis, pericarditis	21	(0.3)	64	(0.3)	104	(0.3)	82	(0.2)	2	(0.5)	1	(0.1)	4	(0.1)	8	(0.3)	13	(0.2)	3	(0.2)
	Hypertensive heart disease	4	(0.1)	27	(0.1)	63	(0.2)	84	(0.2)	0	(0.0)	1	(0.1)	5	(0.2)	13	(0.4)	15	(0.2)	5	(0.3)
	Atrial fibrillation or flutter	108	(1.7)	503	(2.2)	1,133	(3.1)	1,177	(3.1)	4	(0.9)	27	(1.7)	60	(2.2)	62	(2.1)	112	(1.8)	41	(2.3)
	Stroke*	277	(4.3)	1,331	(5.8)	2,576	(7.0)	3,010	(8.0)	10	(2.4)	70	(4.5)	139	(5.0)	184	(6.3)	325	(5.4)	69	(3.9)
	Kidney Failure	73	(1.1)	224	(1.0)	394	(1.1)	365	(1.0)	2	(0.5)	17	(1.1)	27	(1.0)	29	(1.0)	64	(1.1)	14	(0.8)
Death		636	(9.9)	2,497	(10.8)	5,191	(14.1)	6,969	(18.6)	22	(5.2)	106	(6.8)	268	(9.7)	451	(15.5)	679	(11.2)	147	(8.4)

COPD, chronic obstructive pulmonary disease.

^**a**^The sum of types A and B is not equal to the total number of patients, due to missing information on influenza virus type that was excluded from this analysis, and patients co-detected with both types of influenza viruses contributed data to both groups.

^*^ chi-squared test for trend: p <0.001 in CDI and p <0.05 in LCI for each complication.

A total of 15,293 (14.7%) CDI and 847 (11.1%) LCI patients died during hospitalization. The percentage of in-hospital deaths increased with age in CDI and in LCI cases from 9.9% and 5.2% in patients aged 60–64 years to 18.6% and 15.5% in those aged ≥85 years, respectively (p < 0.001, both, Table 4). Complications were similar irrespective of influenza type for all disease categories. However, the percentage of in-hospital deaths associated with influenza type A was 11.2%, significantly higher when compared to 8.4% associated with influenza B (p < 0.001) (Table 4).

Detailed frequencies and proportions of acute heart failure, stroke, and in-hospital death during hospitalization in inpatients with CDI and LCI by season can be found in S5 Table.

### Use of medical resources

The median of length of hospital stays in inpatients with CDI and LCI increased with age (CDI, r^2 = 0.01, p <0.001; LCI r^2 = 0.01, p <0.001) (Table 5). Oxygen therapy use in CDI and in LCI increased with age from 9.4% and 3.3% in patients aged 60–64 years to 14.7% and 10.7% in those aged ≥ 85 years, respectively (p <0.001, both). In contrast, mechanical ventilation used in CDI decreased with age from 2.4% in those aged 60–64 years to 1.6% in patients aged ≥ 85 years (p <0.001). The proportions of dialysis, blood transfusion, and ICU admission in CDI and LCI decreased with age (p <0.001, all). No obvious difference was found in resource consumption by influenza virus type (Table 5).

**Table 5:** Medical resource consumption in inpatients with clinically diagnosed and laboratory confirmed influenza.

Medical resource n, (%)	Clinically diagnosed influenza								Laboratory-confirmed influenza											
	Age group								Age group								Influenza type*			
	60–64		65–74		75–84		85-		60–64		65–74		75–84		85-		Type A		Type B	
	(n = 6,440)		(n = 23,018)		(n = 36,773)		(n = 37,485)		(n = 425)		(n = 1,551)		(n = 2,777)		(n = 2,901)		(n = 6,062)		(n = 1,760)	
Length of hospital stay Median days, (IQR)*	18.0 (9.0, 45.0)		19.0 (10.0, 47.0)		22.0 (11.0, 57.0)		25.0 (13.0, 62.0)		17.0 (8.0, 42.0)		20.0 (9.0, 50.5)		21.0 (9.0, 55.0)		27.0 (11.0, 65.0)		23.0 (10.0, 59.0)		20.0 (9.0, 47.0)	
	N	(%)	n	(%)	n	(%)	n	(%)	n	(%)	n	(%)	n	(%)	n	(%)	n	(%)	n	(%)
Oxygen therapy^	604	(9.4)	2,506	(10.9)	4,620	(12.6)	5,510	(14.7)	14	(3.3)	115	(7.4)	203	(7.3)	311	(10.7)	525	(8.7)	117	(6.6)
Mechanical ventilations	154	(2.4)	495	(2.2)	799	(2.2)	584	(1.6)	6	(1.4)	20	(1.3)	31	(1.1)	26	(0.9)	67	(1.1)	17	(1.0)
Dialysis^#^	278	(4.3)	1,021	(4.4)	1,063	(2.9)	465	(1.2)	22	(5.2)	78	(5.0)	75	(2.7)	40	(1.4)	155	(2.6)	52	(3.0)
Tube feeding	59	(0.9)	202	(0.9)	414	(1.1)	342	(0.9)	3	(0.7)	13	(0.8)	24	(0.9)	26	(0.9)	50	(0.8)	17	(1.0)
Blood transfusion^#^	651	(10.1)	2,383	(10.4)	3,495	(9.5)	2,872	(7.7)	22	(5.2)	121	(7.8)	138	(5.0)	130	(4.5)	326	(5.4)	92	(5.2)
ICU admission^	407	(6.3)	1,513	(6.6)	2,042	(5.6)	1,604	(4.3)	17	(4.0)	91	(5.9)	97	(3.5)	76	(2.6)	223	(3.7)	92	(5.2)

^**a**^The sum of types A and B is not equal to the total number of patients, due to missing information on influenza virus type that was excluded from this analysis, and patients co-detected with both types of influenza viruses contributed data to both groups.

^*^ Spearman’s rank correlation coefficient: p <0.001 for both CDI and LCI.

^chi-squared test for trend: p <0.001 for both CDI and LCI.

^**#**^ chi-squared test for trend (inverse trend): p <0.001 for both CDI and LCI.

IQR; interquartile range, ICU; intensive care unit.

## Discussion

Our study suggests that influenza could represent, on average, 0.5% to 6% of all annual hospitalizations in Japan among people ≥60 years, proportions which increased with age. Outpatient care visits potentially associated with influenza were 2- to 3-times more common than hospitalizations. Although the majority of medically-attended influenza (both CDI and LDI) occurred in people with existing comorbidities, patients who did not have any known underlying chronic conditions represented around a third of outpatients and over 10% of inpatients with laboratory confirmed infection, respectively, a reminder that influenza can also be severe in otherwise healthy older adults [8].

Among LCI cases, we identified the contribution of influenza virus types A and B which co-circulate annually in varying degrees of intensity. In recent years, influenza B viruses have caused substantial epidemics because of evolutionary and epidemiological viral changes [22]. In Japan influenza B viruses were contributed substantially to the seasonal epidemics in 2013/2014, 2015/2016, and 2017/2018 [23–25]. The frequency of complications and resource utilization were similar between patients diagnosed with influenza virus types A and B, which is consistent with previous publications [26–29]. Although we did not have access to viral strain data, influenza B Yamagata and Victoria lineages have been co-circulating since late 1980’s [30], supporting the recommendation for an influenza vaccine that covers both lineages [31].

We identified differences between LCI and CDI, diagnoses of which were more broadly spread throughout the year. In Japan, virus isolation and RT-PCR are mostly conducted in laboratory settings or for research purposes and are rarely a component of routine care. For the diagnosis of influenza, a variety of IRDTs, which are easy, rapid, and low cost, are currently widely used in Japan, and likely the most commonly-used influenza confirmatory tests captured in our study. The sensitivity of IRDTs for seasonal influenza B viruses is lower or similar compared to that of seasonal influenza A viruses [32]. The reported sensitivities of IRDTs for influenza viruses in general ranges between 10%–80%, with lower sensitivity observed among older adults and in those with underlying conditions [14,33–35]. Specific data on the sensitivity of IRDTs in the Japanese population are scarce, but we should assume a proportion of false-negative results, particularly considering the ageing Japanese population, which could lead to underestimation of disease burden. Conversely, CDI likely includes a proportion of false-positive influenza diagnoses following respiratory infection caused by other pathogens in individuals who never received a confirmatory test. By using two different definitions for influenza we hope to represent the possible range of the full impact of seasonal influenza on older adults and on the Japanese health care system.

In our study, the most frequently observed complications were myopathy, respiratory failure and those associated with the cardiovascular system. Among those with LCI, acute heart failure was documented across age groups with a frequency of 4.9 to 8.0%, and stroke varying from 2.4 to 6.3%; both increasing with age. The contribution of influenza virus infection to acute heart failure, stroke, and myocardial infarction was reported as approximately 6%, 0.2%, and 6%, respectively, in Western settings [36,37]. Piroth L et al. recently reported that patients admitted to hospital with COVID-19 more frequently developed acute respiratory failure (27.2%), pulmonary embolism, septic shock, or hemorrhagic stroke than patients with influenza, but less frequently developed myocardial infarction or atrial fibrillation [38]. Systemic inflammatory response, oxidative stress, and activation of prothrombotic pathways may increase acute myocardial infarction documented as potentially associated with influenza [39–41]. Although direct associations between acute heart failure and influenza have not been well characterized [42], acute heart failure-associated hospitalizations correlate with influenza activity [43,44], and therefore, guidelines recommend influenza vaccination for patients at risk of heart failure. Other, non-respiratory, related complications documented in our patient population were myopathy, which also increased with age, being highest among those ≥85 years in the CDI group (17.6%) and in the LCI group (14.1%). Myopathy could be associated with the longer length of hospital stay among this age group and was observed in similar rates among patients with influenza A and B. Rhabdomyolysis has been described as a complication of influenza [45], but we did not have enough clinical information to explore why myopathy was so prevalent in this patient population which may warrant further investigations. The presence of associated comorbidities in older adults has been shown to increase the odds of ICU admission, severe and fatal infections following influenza infection by up to 7-fold with particular risk associated with immunodeficiency, respiratory disease and chronic liver disease identified in different population groups. [46,47] Determining the independent contribution of age from other risk factors is complicated by interactions and confounding treatment practices, but a Spanish study identified an elevated risk of death in older adults aged >65 years; but a lower risk of ICU admission in adults aged >75 years, in comparison with younger adults [48]. Targeted studies to identify populations at highest risk of poor outcome following influenza infection in Japan would help to focus and ensure optimal vaccine coverage in those most at-risk, and improve understanding of the role of comorbidities vs the pathophysiological impact of influenza infection, in complicated hospitalizations.

The observed in-hospital mortality in our study was higher than that reported from a Japanese cohort of 579 adult hospitalized patients (overall 4% death within 30 days follow-up) [6]. This difference could be attributed to the age distribution of patients (younger in the previous study), the type of clinical management received, or due to their study small sample size. Piroth et. al., reported that, in France, in-hospital mortality associated with influenza was ~6%, and that increased to 18% among patients admitted to the ICU and increased with age because of differences in healthcare systems, threshold for hospitalization and ICU admission, underlying health characteristics of the various populations, and how the data are presented (which in most cases do not report by small age groups). Influenza vaccination is the cornerstone to prevent influenza in the community, and despite low to moderate vaccine effectiveness documented from year to year, there are vaccines that can afford better protection to high-risk populations and the elderly but they are not yet available in Japan [49–52].

Finally, we found an increase in healthcare resource utilization by age illustrated by the prolonged length of hospital stay and use of oxygen therapy among the oldest age groups. Nonetheless, ICU admissions, mechanical ventilation, blood transfusion, and dialysis were more frequent among the younger age groups, which could indicate presence of comorbidities or a more parsimonious approach to invasive interventions when dealing with the older patients [53,54].

The present study showed that the proportion of influenza-related all-cause hospitalizations was increasing annually. In the HIS conducted by NIID, the number of influenza hospitalizations in those aged ≥60 years doubled after 2016/2017 [17–20]. Our LCI data matched the relative rate reported based on HIS data between 2011/2012 and 2015/2016. There is no data on 2010/2011 in HIS, however, the estimated number of patients aged 60 years and older with medical visit for influenza in 2010/2011 was less than half of those in 2011/2012 [52]. The consistency of different study results may suggest the legitimacy of our data in the 2011/2012 season but additional investigations to explain the increasing trend, particularly into the frequency and criteria for confirmatory diagnostic use, are warranted.

### Limitations

Several limitations merit consideration in our study. First the 77 enrolled facilities may not be fully representative of the whole healthcare system in Japan and despite its wide distribution, they may not be geographically presentative of the Japanese population. In particular, small hospitals are severely under-represented in the RWD database, potentially biasing results based on their different patient population characteristics and frequency of diagnostic use. This possibility should be investigated with additional studies actively enrolling patients at smaller health facilities. Nonetheless, it still provides insights on the potential impact of influenza to this age group and to the healthcare system, signaling the need for further studies to delineate the burden of influenza in Japan. Similarly, as the number of enrolled medical institutions increased; the proportion of patients aged between 60 to 64 years with CDI and LCI decreased; and the proportion of patients aged ≥85 years relatively increased; over the study period. This might be partially explained by the fact that the institutions added to the RWD database in recent years may admit more elderly patients, who are more likely to be diagnosed with influenza. The change of age distribution in the current database may also reflect the aging population in Japan but it is likely that at least a proportion of the observed increase in influenza over time was associated with the aging population included in the study, and a higher likelihood of influenza diagnostic use in older adults. Similar increases in influenza hospitalization were observed in national surveillance over the study period, possibly stemming from increased frequency of diagnostic use, but we did not have access to information on patients testing negative to assess this. The use of a total hospitalized denominator risks the incorrect impression of variable influenza activity based on the overall hospitalization rate, and real-world databases capturing population denominators would be a useful resource for future studies.

Finally, we did not have access to information such as vaccination records, nursing home residents’ registries, and underlying frailty which could have aid in the interpretation of our results. Lack of antiviral prescription data potentially led underestimation of the morbidity and mortality of the diseases. Lack of antiviral prescription data potentially led underestimation of the morbidity and mortality of the diseases. Moreover, the lack of longitudinal data may also lead to overlooking or underestimating severe complications after patients were transferred to other medical institutions.

## Conclusion

Influenza has an important impact on adults ≥60 years in Japan, and may represent up to 20% of annual hospitalizations during influenza seasons. This leads to a substantial use of medical resources, especially because this population is at risk of influenza related complications or decompensation of underlying chronic medical conditions. Considering the burden associated with medically-attended influenza in this population, influenza vaccination should be emphasized by general practicians as well as specialists like cardiologists to protect this aging population. Vaccines that can afford improved protection in this group could have a large impact on reducing healthcare utilization and the risk for severe diseases and complications associated with influenza.

## Supporting information

S1 Table Codes of the comorbidities used in the study. (DOCX)

S2 Table Codes of the complications used in the study. (DOCX)

S3 Table Procedure codes used in the study. (DOCX)

S4 Table Distribution of influenza virus type among medically-attended patients with laboratory-confirmed influenza, by influenza season (2010/2011–2018/2019). (DOCX)

S5 Table Complications of acute heart failure, stroke, and death in inpatients with clinically diagnosed and laboratory-confirmed influenza. (DOCX)
