# Supplementary material for: Seasonal influenza, its complications and related healthcare resource utilization among people 60 years and older: A descriptive retrospective study in Japan
Source: PLoS One. 2022 Oct 3;17(10):e0272795. doi: 10.1371/journal.pone.0272795 (PMC9529100; doi:10.1371/journal.pone.0272795)
Supplement: S1 Table — (DOCX) [file pone.0272795.s001.docx]

S1 Table. Codes of the comorbidities used in the study

| Disease | ICD-10^a^ |
| --- | --- |
| Chronic lung disease | A15 - A19, D86, J40-J47, J84 |
| Asthma | J45 |
| Diabetes | E10–E14 |
| Neurological disease | G20–G22, G30 - G32, I63, I69.3 |
| Hypertensive disease | I10-I13 |
| Chronic heart failure | I50.9 |
| Chronic heart disease | I05-I09, I20-I25, I26-28, I30-I52, Q20-Q28, Z95 |
| Chronic liver disease | K70 – K77 |
| Chronic kidney disease | N03-N06, N18, N19 |
| End stage renal disease | N18.5 |
| Cancer | C00-C97 |
| Anemia | D50-D53, D55-D59 |

^a^The International Statistical Classification of Diseases and Related Health Problems 10^th^ Revision.
