# Supplementary material for: Seasonal influenza, its complications and related healthcare resource utilization among people 60 years and older: A descriptive retrospective study in Japan
Source: PLoS One. 2022 Oct 3;17(10):e0272795. doi: 10.1371/journal.pone.0272795 (PMC9529100; doi:10.1371/journal.pone.0272795)
Supplement: S2 Table — (DOCX) [file pone.0272795.s002.docx]

S2 Table. Codes of the complications used in the study

| Disease | ICD-10^a^ |
| --- | --- |
| Respiratory failure | J96 |
| Exacerbations of chronic obstructive pulmonary disease | J44.1 |
| Asthma exacerbation | J46 |
| Exacerbations of diabetes | E11.0, E11.1, E12.0, E12.1, E13.0, E13.1, E14.0, E14.1 |
| Encephalitis | A85.8, A86, A87.8, A87.9, A89, B94.1, G03.8, G03.9, G04.0, G04.8, G04.9, G05.1, G05.8, G36 |
| Myopathy | M62.5, M62.8, M62.9 |
| Myositis | M60 |
| Hypertensive disease | I10 – I13 |
| Acute myocardial infarction | I21 – I24 |
| Acute heart failure | I50 |
| Hypertensive heart failure | I11.0, I13.9, I50.9 |
| Myocarditis and pericarditis | B33.2, I30, I31.9, I40, I41.1, I51.4, I51.8 |
| Hypertensive heart disease | I11.0, I11.9, I13.9 |
| Atrial fibrillation or flutter | I48 |
| Stroke | I60 – I69 |
| Kidney failure | N17, I12 |

^a^The International Statistical Classification of Diseases and Related Health Problems 10th Revision.
