# Supplementary material for: Seasonal influenza, its complications and related healthcare resource utilization among people 60 years and older: A descriptive retrospective study in Japan
Source: PLoS One. 2022 Oct 3;17(10):e0272795. doi: 10.1371/journal.pone.0272795 (PMC9529100; doi:10.1371/journal.pone.0272795)
Supplement: S3 Table — (DOCX) [file pone.0272795.s003.docx]

S3 Table. Procedure codes used in the study

| Procedure | Domestic procedural code |
| --- | --- |
| Intensive care unit | 190024710, 190066710, 190066810, 190116310, 190116410, 190117310, 190120810, 190139810, 190139910, 190140010, 190140110, 190140270, 190140370, 190140410, 190140510, 190149910, 190150010, 190717210, 190717310, 190717410, 190717510, 190717610, 190717710, 190717810, 190717910, 190718010, 190718110, 190718210, 190718310, 190718410, 190718510, 190718610, 190727710, 190727810, 190727910, 190728010, 190728110, 190728210, 190728310, 190728410, 190728510, 190728610, 190728710, 190728810, 190728910, 190729010, 190729110, 190729210, 190729310, 190729410, 190729510, 190729710, 190730010, 190730310, 190730610, 190730810, 190730910, 190731010, 190737210, 190737310, 190737410, 190737510, 190737610, 190737710, 190737810, 190737910, 190738010, 190738110, 190738210, 190738310, 190738410, 190738510, 190738610, 190738710, 190738810, 190738910, 190739010, 190739210, 190739510, 190739810, 190740110, 190740310, 190740410, 190740510, 190746710, 190746810, 190746910, 190747010, 190747110, 190747210, 190747310, 190747410, 190747510, 190747610, 190747710, 190747810, 190747910, 190748010, 190748110, 190748210, 190748310, 190748410, 190748510, 190748710, 190749010, 190749310, 190749610, 190749810, 190749910, 190750010, 193001610, 193001710, 193001810, 193001910, 193002010, 193002110, 193002210, 193002310, 193002410, 193003010, 193003110, 193003210, 193005910, 193006010, 193006110, 193006210, 193006310, 193006410, 193006570, 193006670, 193006710, 193006810, 193006910, 193007010, 193007110, 193007210, 193009210, 193009310, 193301410, 193301510, 193301810, 193301910, 193302010, 193302110, 193302210, 193302310, 193302410, 193302510, 193302610, 193302710, 193307210, 193307310, 193307410, 193307510, 193307610, 193307710, 193307870, 193307970, 193308010, 193308110, 193308210, 193308310, 193308410, 193308510, 193309210, 193309310, 193501610, 193501710, 193501810, 193501910, 193502010, 193502110, 193502210, 193502310, 193502410, 193502510, 193502610, 193504510, 193507510, 193507610, 193507710, 193507810, 193507910, 193508010, 193508170, 193508270, 193508310, 193508410, 193508510, 193508610, 193508710, 193508810, 193509510, 193509610, 190174410, 190174510, 190174610, 190174710, 190174810, 190174910, 190175010, 190175110, 193010010, 193010110, 193010210, 193010310, 193010410, 193010510, 193010610, 193010710, 193010810, 193010910, 193011010, 193011110, 193310210, 193310310, 193310410, 193310510, 193310610, 193310710, 193310810, 193310910, 193311010, 193311110, 193311210, 193311310, 193511710, 193511810, 193511910, 193512010, 193512110, 193512210, 193512310, 193512410, 193512510, 193512610, 193512710, 193512810, 193011310, 193011410, 193313110, 193313210, 193514010, 193514110, 190207870 |
| Mechanical ventilation | C107, C164, C164, C164, J026-3, J045, A306, A309, A307 |
| Oxygen therapy | C157, J024, J025, J045, J115, J045 |
| Dialysis | 140007710, 140007910, 140008170, 140029850, 140033770, 140036710, 140051010, 140051110, 140052570, 140052810, 140052970, 140053670, 140055970, 140057810, 140057910, 140058010, 140058110, 140058210, 140058310, 140058410, 140058510, 140058610, 140058770, 140058870, 140058970, 140059070, 140059170, 140059270, 140059310, 140059410, 140059510 |
| Blood transfusion | 150224810, 150224910, 150225010, 150225110, 150225210, 150225310, 150225410, 150225510, 150225610, 150225770, 150225850, 150247010, 150247110, 150254810, 150278910, 150286210, 150286310, 150286410, 150286510, 150287450, 150327510, 150327610, 150327710, 150327810, 150327910, 150328010, 150366370, 150366470, 150366570, 150366670, 150380070, 150390610, 150390710, 150404970 |
| Tube feeding | J043-4, J120 |
