# Supplementary material for: Seasonal influenza, its complications and related healthcare resource utilization among people 60 years and older: A descriptive retrospective study in Japan
Source: PLoS One. 2022 Oct 3;17(10):e0272795. doi: 10.1371/journal.pone.0272795 (PMC9529100; doi:10.1371/journal.pone.0272795)
Supplement: S4 Table — (DOCX) [file pone.0272795.s004.docx]

S4 Table. Distribution of influenza virus type among medically-attended patients with laboratory-confirmed influenza, by influenza season (2010/2011 - 2018/2019).

|  |  | Total | | Season | | | | | | | |
| --- | --- | --- | --- | --- | --- | --- | --- | --- | --- | --- | --- |
|  |  |  |  | 2010/2011 | | 2011/2012 | | 2012/2013 | | 2013/2014 | |
| Number of laboratory-confirmed influenza | | 31,122 | | 819 | | 1,738 | | 2,548 | | 1,892 | |
|  |  | n | (%) | n | (%) | n | (%) | n | (%) | n | (%) |
| Type of influenza | | | | | | | | | | |  |
|  | Type A | 24,515 | (78.8) | 716 | (87.4) | 1,468 | (84.5) | 2,340 | (91.8) | 1,278 | (67.5) |
|  | Type B | 8,321 | (26.7) | 163 | (19.9) | 406 | (23.4) | 387 | (15.2) | 787 | (41.6) |
|  | Unknown | 1,075 | (3.5) | 40 | (4.9) | 66 | (3.8) | 90 | (3.5) | 59 | (3.1) |
|  | Type AB | 2,789 | (9.0) | 100 | (12.2) | 202 | (11.6) | 269 | (10.6) | 232 | (12.3) |
|  |  | Season | | | | | | | | | |
|  |  | 2014/2015 | | 2015/2016 | | 2016/2017 | | 2017/2018 | | 2018/2019 | |
| Number of laboratory-confirmed influenza | | 3,990 | | 3,368 | | 5,369 | | 6,190 | | 5,208 | |
|  |  | n | (%) | n | (%) | n | (%) | n | (%) | n | (%) |
| Type of influenza | | | | | | | | | | |  |
|  | Type A | 3,547 | (88.9) | 2,030 | (60.3) | 4,942 | (92.0) | 3,177 | (51.3) | 5,017 | (96.3) |
|  | Type B | 619 | (15.5) | 1,534 | (45.5) | 605 | (11.3) | 3,372 | (54.5) | 448 | (8.6) |
|  | Unknown | 198 | (5.0) | 81 | (2.4) | 197 | (3.7) | 192 | (3.1) | 152 | (2.9) |
|  | Type AB | 374 | (9.4) | 277 | (8.2) | 375 | (7.0) | 551 | (8.9) | 409 | (7.9) |

The sum of A, B, unknown and AB is not equal to the total number of patients, because the patients whom both types of influenza viruses are co-detected contributed to both type A and B groups.
