# Supplementary material for: Seasonal influenza, its complications and related healthcare resource utilization among people 60 years and older: A descriptive retrospective study in Japan
Source: PLoS One. 2022 Oct 3;17(10):e0272795. doi: 10.1371/journal.pone.0272795 (PMC9529100; doi:10.1371/journal.pone.0272795)
Supplement: S5 Table — (DOCX) [file pone.0272795.s005.docx]

**S5 Table. Complications of acute heart failure, stroke, and death in inpatients with clinically diagnosed and laboratory-confirmed influenza**

|  | | | Season | | | | | | | | | Total |
| --- | --- | --- | --- | --- | --- | --- | --- | --- | --- | --- | --- | --- |
|  | | | 2010/2011 | 2011/2012 | 2012/2013 | 2013/2014 | 2014/2015 | 2015/2016 | 2016/2017 | 2017/2018 | 2018/2019 |  |
| All-cause hospitalization (n) | | | 137,854 | 148,732 | 164,522 | 181,727 | 203,051 | 231,148 | 239,229 | 234,202 | 222,782 | 1,763,247 |
| Clinically diagnosed influenza | | | 5,128 | 5,575 | 6,551 | 7,788 | 10,865 | 12,463 | 18,037 | 18,972 | 18,337 | 103,716 |
|  | Acute heart failure (n) | | 567 | 595 | 683 | 784 | 1,112 | 1,224 | 1,671 | 1,627 | 1,342 | 9,605 |
|  |  | /all-cause hospitalization (‰) | (4.11) | (4.00) | (4.15) | (4.31) | (5.48) | (5.30) | (6.98) | (6.95) | (6.02) | (5.45) |
|  |  | /CDI hospitalization (%) | (11.1) | (10.7) | (10.4) | (10.1) | (10.2) | (9.8) | (9.3) | (8.6) | (7.3) | (9.3) |
|  | Stroke (n) | | 412 | 454 | 513 | 566 | 782 | 1,010 | 1,302 | 1,233 | 922 | 7,194 |
|  |  | /all-cause hospitalization (‰) | (2.99) | (3.05) | (3.12) | (3.11) | (3.85) | (4.37) | (5.44) | (5.26) | (4.14) | (4.08) |
|  |  | /CDI hospitalization (%) | (8.0) | (8.1) | (7.8) | (7.3) | (7.2) | (8.1) | (7.2) | (6.5) | (5.0) | (6.9) |
|  | Death (n) | | 865 | 917 | 1,064 | 1,165 | 1,643 | 1,801 | 2,611 | 2,768 | 2,459 | 15,293 |
|  |  | /all-cause hospitalization (‰) | (6.27) | (6.17) | (6.47) | (6.41) | (8.09) | (7.79) | (10.91) | (11.82) | (11.04) | (8.67) |
|  |  | /CDI hospitalization (%) | (16.9) | (16.4) | (16.2) | (15.0) | (15.1) | (14.5) | (14.5) | (14.6) | (13.4) | (14.7) |
| Laboratory-confirmed influenza | | | 212 | 488 | 697 | 438 | 897 | 768 | 1,404 | 1,479 | 1,271 | 7,654 |
|  | Acute heart failure (n) | | 16 | 18 | 38 | 32 | 53 | 54 | 88 | 111 | 67 | 477 |
|  |  | /all-cause hospitalization (‰) | (0.12) | (0.12) | (0.23) | (0.18) | (0.26) | (0.23) | (0.37) | (0.47) | (0.30) | (0.27) |
|  |  | /LCI hospitalization (%) | (7.5) | (3.7) | (5.5) | (7.3) | (5.9) | (7.0) | (6.3) | (7.5) | (5.3) | (6.2) |
|  | Stroke (n) | | 14 | 20 | 49 | 26 | 45 | 41 | 80 | 66 | 62 | 403 |
|  |  | /all-cause hospitalization (‰) | (0.10) | (0.13) | (0.30) | (0.14) | (0.22) | (0.18) | (0.33) | (0.28) | (0.28) | (0.23) |
|  |  | /LCI hospitalization (%) | (6.6) | (4.1) | (7.0) | (5.9) | (5.0) | (5.3) | (5.7) | (4.5) | (4.9) | (5.3) |
|  | Death (n) | | 23 | 45 | 66 | 50 | 110 | 86 | 163 | 153 | 151 | 847 |
|  |  | /all-cause hospitalization (‰) | (0.17) | (0.30) | (0.40) | (0.28) | (0.54) | (0.37) | (0.68) | (0.65) | (0.68) | (0.48) |
|  |  | /LCI hospitalization (%) | (10.8) | (9.2) | (9.5) | (11.4) | (12.3) | (11.2) | (11.6) | (10.3) | (11.9) | (11.1) |

CDI, clinically diagnosed influenza; LCI, laboratory-confirmed influenza
